# Distress Intolerance Is Associated With a Greater Reward Positivity to Aversive Avoidance Feedback

**DOI:** 10.1111/psyp.70236

**Published:** 2026-02-04

**Authors:** Brian J. Albanese, Daniela Porro, David H. Johnson, Bryce K. Clausen, Anka A. Vujanovic, Richard J. Macatee

**Affiliations:** ^1^ Department of Psychological and Brain Sciences Texas A&M University College Station Texas USA; ^2^ Department of Psychology Florida State University Tallahassee Florida USA

## Abstract

Research indicates that the reward positivity (RewP) can be elicited by both appetitive gain (e.g., winning money) and aversive outcome avoidance (e.g., safety from a noise blast). Yet, little work has linked these differential reward responses with avoidance‐related psychopathology risk factors. The present study addressed this gap by examining the associations of distress intolerance (DI), a risk factor for maladaptive avoidance‐based strategies, with reward responses during two versions of a simple guessing task: the monetary doors task and aversive avoidance doors task. Young adults (*n* = 102) were recruited from a large university campus to complete two versions of a doors task in which they were instructed to choose one of two doors and view feedback that indicated monetary gain/loss in one task (i.e., monetary doors task) or avoidance/administration of an aversive noise blast in the other task (i.e., aversive avoidance doors task). The RewP was extracted at FCz from 250 to 350 ms in each condition. ΔAvoidance RewP was calculated as the residualized difference score of Avoidance Win (i.e., no aversive sound) relative to Avoidance Loss (i.e., pending noise blast administration). ΔMonetary RewP was calculated as the residualized difference score of Monetary Win relative to Loss. Feedback‐locked P300 waveforms in each condition were also extracted. Results indicated that greater self‐reported DI was linked with a larger ΔAvoidance RewP (*β* = 0.32, *p* = 0.026) and Avoidance Feedback P300 (*β* = 0.36, *p* = 0.012) but not the ΔMonetary RewP (*p* = 0.467) or Monetary Feedback P300 (*p* = 0.573). Findings were not better explained by self‐reported symptoms of depression, trait anxiety, trauma history, or task‐related state anxiety. The present study demonstrates that elevated DI is associated with exaggerated reward activation to avoidance‐related feedback (ΔAvoidance RewP). Taken together, this work advances our understanding of DI and suggests the utility of the ΔAvoidance RewP for understanding disruptions of negative reinforcement.

## Impact Statement

Distress Intolerance (DI) is a well‐researched transdiagnostic vulnerability factor and reflects the belief that negative emotionality cannot be tolerated and should be avoided. The present study leveraged the Reward Positivity (RewP) and Feedback‐locked P300 measured from both appetitive (monetary gain) and a modified aversive stimulus (i.e., noise blast) avoidance doors tasks to demonstrate that DI is marked by greater reward responsivity specifically when the ‘reward’ is avoiding an aversive stimulus. These findings inform our understanding of DI and support the use of the ΔAvoidance RewP to understand avoidance‐related disruptions.

Distress intolerance (DI) is a well‐established cognitive‐affective vulnerability factor that reflects the perceived ability to withstand aversive affective states (Leyro et al. 2010; Vujanovic and Zegel 2020). Abundant research has linked DI with psychopathology spanning internalizing and externalizing conditions (Leyro et al. 2010; Vujanovic and Zegel 2020), including anxiety and mood‐related pathology (Albanese et al. 2021; Allan et al. 2014; Macatee, Albanese, Clancy, et al. 2018; Macatee et al. 2015, 2013), posttraumatic stress disorder (Vujanovic and Zegel 2020), substance use‐related disorders (Bornovalova et al. 2012; Brown et al. 2009; Daughters et al. 2005; Macatee, Albanese, Crane, et al. 2018; Macatee et al. 2019), and personality disorders (Bornovalova et al. 2008, 2011; Daughters et al. 2008; Sargeant et al. 2011). Despite the clear importance of DI in understanding transdiagnostic risk across distress‐related disorders, the underlying neural mechanisms driving DI remain largely unclear, thereby limiting the development and enhancement of interventions to ameliorate elevated DI.

One plausible neural mechanism underlying this risk is neural reactivity to the escape or avoidance of aversive affective states, as theoretical viewpoints on DI posit that individual differences exist in the desire to escape or avoid these states and the situations in which they may arise (Leyro et al. 2010; Vujanovic and Zegel 2020). Abundant empirical work has sought to explicate the mechanisms underlying DI through this lens, largely with the use of behavioral paradigms measuring one's latency to stop a distressing task (Lejuez et al. 2003). For instance, individuals who are high on the DI construct are quicker to stop an aversive task and therefore cease the associated negative emotionality (see Leyro et al., for a review), though some studies fail to find convergence across self‐report and behavioral measures of DI (e.g., McHugh et al. 2011; Vujanovic et al. 2017, 2018). This perhaps suggests the need for the utilization of alternative methods in order to understand how escape/avoidance of affective states contributes to DI.

Another way to index individual differences in the proclivity to avoidance is to leverage well‐established appetitive reward‐related paradigms to reflect sensitivity to aversive stimulus avoidance (LeDoux et al. 2017). Ample evidence suggests that aversive avoidance activates canonical mesolimbic reward circuitry and elicits similar behaviors across species as appetitive rewards (Smith and Buchanan 1954; Tanimoto et al. 2004), but that aversive stimulus avoidance remains conceptually distinct from appetitive reward (LeDoux et al. 2017). Considering that appetitive reward has shown robust individual differences (e.g., Proudfit 2015; Van den Berg et al. 2011), it is plausible that these paradigms could be modified to reflect individual differences in avoidance‐based reward and, therefore, provide insights into avoidance‐related constructs such as DI.

Among the most well‐researched indices of reward‐related neural activation is the reward positivity (RewP; Proudfit 2015). The RewP is an event‐related potentials (ERPs) that reflects individual differences in the initial response to rewards. The RewP is the most commonly analyzed reward‐based component and is typically maximal at frontocentral electrode sites (e.g., FCz) approximately 250–350 ms following feedback indicating rewards, relative to non‐rewards (see Proudfit 2015 for a review). The RewP has been traditionally used to understand appetitive reward and is most commonly elicited using a simple monetary game in which participants make choices and then view feedback indicating that they earned an appetitive reward (e.g., money, nicotine) or feedback indicating either a loss or non‐reward (Baker et al. 2016; Foti and Hajcak 2009; Holroyd et al. 2008; Proudfit 2015). The RewP has primarily been studied in the context of depression, with the majority of research showing a blunted RewP is associated with low positive emotionality (Kujawa et al. 2015) and depression (Belden et al. 2016; Brush et al. 2018; Foti and Hajcak 2009; Liu et al. 2014). Further, the RewP prospectively predicts the onset of depression (Bress et al. 2013; Nelson et al. 2016) and amplifies the effects of stress on subsequent depression in adolescent girls (Burani et al. 2019). Importantly, research has shown that the reward vs. non‐reward difference is driven by variability in the response to rewards (Foti et al. 2011), and the RewP has shown good psychometric properties (e.g., Levinson et al. 2017; Luking et al. 2017), thereby further supporting the use of this ERP to index reward responses. In addition to the RewP, some studies also examine the feedback‐related P300, which is typically measured from approximately 250–450 ms at centroparietal sites and indexes attentional allocation to salient stimuli (e.g., rewards; Hajcak and Foti 2020).

As suggested by Albanese and Hajcak (2021), the RewP could be leveraged to study individual differences in aversive avoidance‐related brain activity by modifying the outcomes in the doors task often used to elicit the RewP. More specifically, a modified doors task in which the “good” outcome reflects avoidance of an aversive stimulus and the “bad” outcome reflects pending administration of an aversive stimulus, with the difference in neural reactivity between these conditions reflecting individual differences in the initial response to avoidance (i.e., *ΔAvoidance RewP*) rather than the typical outcomes of winning/losing money (i.e., *ΔMonetary reward RewP*). In fact, several studies have utilized similar paradigms with success, as modified doors tasks have both demonstrated the phenomenology of the RewP as a reward‐related component that is not simply salience driven (Bauer et al. 2024; Heydari and Holroyd 2016; Mulligan and Hajcak 2018) and have been used to examine predictors of problematic cannabis use (Preston et al. 2022). Thus, the aversive avoidance RewP may represent a promising new avenue for studying individual differences in reward‐related constructs (Albanese and Hajcak 2021), including DI (Leyro et al. 2010; Vujanovic and Zegel 2020).

The current study was designed to evaluate reward‐related neural responding to the avoidance of aversive affective outcomes along the continuum of DI and to provide initial evidence for the specificity of the ΔAvoidance RewP and feedback‐related P300 to understanding avoidance‐related disruptions. To accomplish this, participants were recruited across the full spectrum of DI to provide ample variability across the entire range of the construct. Participants completed a modified doors gambling task in which the winning outcome represented avoidance of an aversive sound, and the losing outcome signaled the pending administration of the aversive sound (Albanese and Hajcak 2021; Mulligan and Hajcak 2018). A classical monetary gain/loss doors task was also included to evaluate the specificity of elevated DI to negative reinforcement‐related disruptions and not general reward alterations. Considering the theoretical foundations of DI and empirical support linking DI with avoidance‐related behaviors (e.g., Lejuez et al. 2003; Leyro et al. 2010; Vujanovic and Zegel 2020), we hypothesized that individuals with high DI would demonstrate a greater aversive avoidance RewP, even when accounting for depression, anxiety, trauma history, and state anxiety during the modified aversive avoidance doors task. We also hypothesized that there would be no differences in the monetary reward responses. Exploratory analyses also examined the feedback‐related P300 in each condition to determine if effects were driven by broader attentional allocation to reward feedback.

## 1 Method

### 1.1 Participants

Participants consisted of emerging adults (*n* = 111) recruited from a large southern university and the surrounding community. To allow for sufficient variability and avoid a restriction of range in the constructs of interest (i.e., DI and aversive avoidance reward responsivity), inclusion criteria included being at least 18 years old, and exclusion criteria were self‐reports of a bipolar or other psychotic‐spectrum disorder. A total of 9 participants were removed from this analysis due to technical issues (*n* = 1), behavioral observations (e.g., falling asleep; *n* = 2), and outlier split‐half residualized difference scores (e.g., monetary RewP even trials regressed upon monetary RewP odd trials) indicating particularly poor reliability (*n* = 6). This yielded a final sample of 102 participants aged 18–23 years old (M = 18.59, SD = 1.00) who identified as predominantly female (*n* = 67; 65.7%) and Caucasian/White (*n* = 69; 67.6%), Black/African‐American (*n* = 5; 4.9%), Native American or Alaskan Native (*n* = 3; 2.9%), Asian or Asian‐American (*n* = 12; 11.8%), or ‘Other’ or more than one race (*n* = 13; 12.7%). Further, over one‐third of participants identified as Hispanic or Latinx (*n* = 37; 36.3%).

### 1.2 Procedures

Interested participants completed a brief screener to determine eligibility and then attended a baseline visit during which verbal and written explanations of the study procedures were provided, and informed consent was obtained. Participants then completed self‐report measures, neural and peripheral electrodes were placed, and participants completed a series of computerized tasks, including the modified doors task described below. All study procedures were approved by the university Institutional Review Board, and participants were compensated with $5 and course credit at the conclusion of their participation.

### 1.3 Measures

#### 1.3.1 Distress Intolerance Index (DII; McHugh and Otto 2012)

The DII is a 10‐item self‐report measure used to assess the perceived inability to tolerate distress and tendency to avoid or escape aversive emotional states (e.g., *I can't handle feeling distressed or upset*; *I'll do anything to stop feeling distressed or upset*). Participants rated how accurately each statement described their experiences on a 5‐point Likert scale ranging from 0 (*very little*) to 4 (*very much*), and items were summed into a total score such that larger scores reflect greater DI. In the current study, the DII demonstrated excellent internal consistency (*α* = 0.94).

#### 1.3.2 Depression, Anxiety, and Stress Scale −21 (DASS‐21; Antony et al. 1998)

The DASS‐21 is a shortened, 21‐item self‐report measure adapted from the original 42‐item DASS (Lovibond and Lovibond 1995). The DASS‐21 indexes depression, anxiety, and overall stress severity over the past week. Participants rate the extent to which each statement applies to them using a 4‐point Likert scale from 0 (*Did not apply to me at all*) to 3 (*Applied to me very much, or most of the time*). The DASS‐21 depression (α = 0.89) and anxiety (α = 0.88) subscales were used in the present study and demonstrated good internal consistency.

#### 1.3.3 Life Events Checklist for DSM‐5 (LEC‐5; Weathers et al. 2013)

The LEC‐5 assesses the number and specific types of traumatic experiences that an individual has experienced in their lifetime, as defined by the DSM‐5 PTSD Criterion A. Participants indicate whether they directly experienced, witnessed, or learned about 17 distinct types of traumatic events (e.g., physical assault, sexual assault). In the current sample, 95 participants reported directly experiencing or witnessing an average of 4.17 traumatic event types (SD = 3.15) in their lifetime.

### 1.4 Monetary and Aversive Avoidance Doors Tasks

The doors task is often used to examine differences in neural processing of win vs. loss feedback (Proudfit 2015). Participants were presented with two identical doors on the screen and given directions to select either the left or right door by clicking the left or the right mouse button, respectively. After selecting a door, a fixation cross appeared for 1000 ms followed by a 2000 ms feedback stimulus consisting of either a green up arrow (indicating a win) or a red down arrow (indicating a loss). The feedback stimulus was followed by another 1000 ms fixation cross, after which the participant continued to the next trial at their own pace.

To evaluate differential effects of reward valence, both a monetary reward and a modified relief version of the doors task were included. Neural relief sensitivity was measured using a previously validated modified doors task (Millner et al. 2019) in which participants are presented with two doors and asked to choose which one they believe has the better outcome behind it. After making a choice, the potential threat of a possible aversive noise blast is introduced along with a fixation period lasting 1000 ms. Participants then view feedback (2000 ms) indicating that the potential threat of an aversive (85 dB, 50 ms) white noise blast will be realized (i.e., *avoidance loss trials*) or escaped (i.e., silence; avoidance win trials; Mulligan and Hajcak 2018). To assess the specificity of findings to aversive avoidance reward responses, and not a general reward sensitivity, a monetary reward task was also included. Initial responses to monetary reward were assessed using a similar guessing task (the “Doors” task) as described above, with participants instructed that feedback indicated that they either won (+$0.30) or lost (−$0.15) money (Proudfit 2015). To account for anticipatory effects of a pending salient sound, the monetary gain condition included a positive sound (i.e., a slot machine winning money; International Affective Digital Sounds [IADS] No. 717; Yang et al. 2018) on monetary gain trials and no sound on monetary loss trials.

Consistent with recommendations (Levinson et al. 2017), each version of the task consisted of 64 trials total (32 winning and 32 losing trials) presented over two consecutive blocks. Task type (i.e., aversive avoidance or monetary gain condition) order was counterbalanced to minimize order effects; all blocks within a task type were presented sequentially to avoid cross‐over effects. Different stimuli (thumbs up/thumbs down; arrow up/arrow down) were used for aversive avoidance and monetary doors tasks for each participant and counterbalanced across participants. Spearman‐Brown corrected split‐half reliability estimates for the RewP were as follows: ΔAvoidance RewP (avoidance win trials relative to noise blast trials, *r* = 0.44), Avoidance Win RewP (*r* = 0.90), Avoidance Loss RewP (pending noise blast; *r* = 0.89), ΔMonetary RewP (monetary gain trials relative to monetary loss, *r* = 0.35), Monetary Win RewP (*r* = 0.89), and Monetary Loss RewP (*r* = 0.91). Spearman‐Brown corrected split‐half reliability estimates were as follows: ΔAvoidance Feedback P3 (avoidance win trials relative to noise blast trials, *r* = 0.35), Avoidance Win Feedback P3 (*r* = 0.93), Avoidance Loss Feedback P3 (pending noise blast; *r* = 0.92), ΔMonetary Feedback P3 (monetary gain trials relative to monetary loss, *r* = 0.40), Monetary Win Feedback P3 (*r* = 0.93), and Monetary Loss Feedback P3 (*r* = 0.91).

#### 1.4.1 Task Delivery and Psychophysiology Measurement

A Dell OptiPlex 780 computer with BrainVision Recorder software was used to collect all EEG data. ERP responses were measured via a 32‐channel actiCHamp Plus amplifier connected to a 32‐channel BrainVision actiCap slim (online sampling rate = 1000 Hz). A midline ground (AFz) and online reference electrode (FCz) were used during EEG recording and were re‐referenced to the average of the mastoid sensors (TP9 and TP10) offline. Additional electrodes placed approximately 1 cm above and below the right eye, as well as on the outer canthus of each, measured vertical and horizontal electrooculogram (EOG) activity, respectively. High‐chloride (10%) Abrasive Electrolyte‐Gel was used to fill each electrode and EEG recording was done while impedance values were below 10 kohms.

### 1.5 Data Processing and Reduction

Offline data processing and extraction was conducted using Brain Vision Analyzer—2 (Brain Products LLC, Munich, Germany). Data were first visually examined for bad channels, which were interpolated with nearby sensors as needed. Data were resampled to 500 Hz, bandpass filtered (0.05 Hz—30 Hz, 4th order), re‐referenced to the averaged mastoids (TP9 and TP10), and then FCz was regenerated. Data were segmented into 1000 ms epochs (−200 ms pre‐stimulus to 800 ms post‐stimulus). Ocular artifacts were corrected using the Gratton et al. (1983) method. An automatic artifact‐rejection procedure was conducted in which data from individual channels were rejected if there was a voltage step greater than 50 μV, a difference greater than 300 μV within a 200 ms interval, an amplitude less than −100 μV or greater than 100 μV, or activity less than 0.5 μV within a 100 ms interval. The RewP was then scored using the averaged activity at FCz during the 250–350 ms time window (Proudfit 2015). The feedback‐locked P300 was scored using the averaged activity from 250–450 ms to capture the pull P300 waveform while minimizing spatial overlap with the RewP.

### 1.6 Data Analytic Plan

Data were first examined for normality. Descriptive statistics and correlations were then examined for bivariate relationships among the avoidance RewP, monetary RewP, and DI. Differences in reward responding across condition (aversive avoidance and monetary reward) and trial type (win, loss) were then examined using repeated measures analysis of variance (RM‐ANOVA). Next, an additional 2 (condition) by 2 (trial‐type) RM‐ANOVA was conducted to examine differential reward responding as a function of DI. Significant interactions in the RM‐ANOVA were further probed using multiple linear regression to examine the directionality of effects. Unstandardized residualized difference scores (e.g., ΔAvoidance RewP: avoidance relative to noise blast; ΔMonetary RewP: monetary win relative to loss) were entered as the dependent variable with DII total score entered as an independent variable.^1^ In addition, DASS depression (e.g., Foa et al. 2006; Proudfit 2015; Zvolensky et al. 2010), DASS anxiety (e.g., Foa et al. 2006; Zvolensky et al. 2010), and trauma history (i.e., number of endorsed traumatic event types experienced; Vujanovic et al. 2018) were entered as independent variables to account for their established relationships with DI, reward activity, and avoidance‐related behaviors. Additional multiple linear regressions were conducted with the signal averaged ERPs for gain and loss entered as the dependent variable to establish if results were driven primarily by brain activity on win or loss trials. The same data analysis plan was conducted for feedback‐locked P300.

## 2 Results

### 2.1 Descriptive Statistics and Correlations

Descriptive statistics and bivariate correlations are presented in Table 1. Full correlations among all study variables can be found in the online supplemental materials (see Table S1). Of note, DII total score was significantly correlated with the ΔAvoidance RewP (*r* = 0.27, *p* = 0.007) but not the ΔMonetary RewP (*r* = −0.02, *p* = 0.839). Aversive noise blast sound ratings were not correlated with the ΔAvoidance RewP (*r* = 0.08, *p* = 0.417), and the monetary reward sound was not correlated with the ΔMonetary RewP (*r* = 0.05, *p* = 0.607), indicating that reward responsivity was not accounted for by the aversiveness of the sound. Further, DII total score was not correlated with ratings of the aversive noise blast (*r* = 0.09, *p* = 0.386) or monetary reward sound (*r* = −0.09, *p* = 0.365). In support of the task manipulation, self‐reported state anxiety during the aversive avoidance condition was significantly related to the ΔAvoidance RewP (*r* = 0.23, *p* = 0.024) but state anxiety during the monetary reward condition, in which anxiety was not manipulated, was not significantly related to the ΔMonetary RewP (*r* = 0.10, *p* = 0.299).^2^ Further, the DASS anxiety, DASS depression, and DII scores indicate a non‐clinical sample, though we did observe variability across the spectrum on these variables (see Table 1).

**TABLE 1:** Descriptive statistics and bivariate correlations.

	1	2	3	4	5	6	7	8	9	10	11	12	13	14
1. DII Total	—													
2. DASS Anxiety	0.70***	—												
3. DASS Depression	0.58***	0.64***	—											
4. LEC Trauma Count	0.24*	0.29**	0.20*	—										
5. ∆Avoidance RewP	0.26**	0.13	0.19	−0.00	—									
6. Avoidance Win RewP	0.10	−0.05	−0.08	−0.10	0.52***	—								
7. Avoidance Loss RewP	−0.04	−0.13	−0.20*	−0.11	0.01	0.86***	—							
8. ∆Monetary RewP	−0.02	0.07	−0.03	−0.04	0.23*	0.29**	0.20*	—						
9. Monetary Win RewP	0.00	0.01	−0.12	−0.04	0.25*	0.74***	0.72***	0.54***	—					
10. Monetary Loss RewP	0.01	−0.03	−0.12	−0.02	0.15	0.71***	0.74***	0.04	0.87***	—				
11. Avoidance Win Feedback P300	0.13	−0.01	−0.16	−0.08	0.36***	0.79***	0.71***	0.30**	0.69***	0.63***	—			
12. Avoidance Loss Feedback P300	0.11	−0.03	−0.14	−0.10	0.18	0.76***	0.78***	0.23*	0.63***	0.62***	0.91***	—		
13. Monetary Win Feedback P300	0.04	0.11	−0.10	−0.06	0.22*	0.55***	0.51***	0.46***	0.82***	0.69***	0.73***	0.67***	—	
14. Monetary Loss Feedback P300	0.10	0.09	−0.09	0.05	0.28**	0.56***	0.49***	0.17	0.74***	0.78***	0.72***	0.67***	0.87***	—
Mean	13.91	4.79	4.75	1.78	−0.16	11.71	8.86	0.06	12.82	10.95	13.28	12.75	14.32	14.44
Standard deviation	10.28	5.10	4.59	1.77	3.67	7.18	7.10	3.58	7.19	7.33	6.06	6.63	6.46	6.69
Minimum	0.00	0.00	0.00	0.00	−7.60	−2.12	−3.72	−7.54	−1.60	−3.80	−0.12	−2.59	−1.68	−1.60
Maximum	40.00	20.00	18.00	10.00	8.88	29.93	30.53	11.71	34.01	38.15	28.39	29.01	30.98	30.23
Skew	0.28	1.03	0.95	1.57	0.28	0.59	0.76	0.35	0.62	0.70	0.23	0.25	0.15	0.11
Kurtosis	−1.01	−0.05	0.01	3.66	−0.41	−0.09	0.36	0.10	0.26	1.00	−0.36	−0.47	−0.24	−0.14

Abbreviations: ΔAvoidance RewP, Unstandardized residualized difference representing Avoidance Win (aversive avoidance)—Avoidance Loss (aversive sound); DASS, Depression, Anxiety and Stress Scale; DII, Distress Intolerance Index; LEC‐5, Life Events Checklist for DSM‐5; ΔMonetary RewP, Unstandardized residualized difference representing Monetary Win—Monetary Loss; RewP, Reward Positivity.

*** *p* < 0.001.

** *p* < 0.01.

* *p* < 0.05.

### 2.2 Manipulation Check

#### 2.2.1 Task Ratings

Participants rated each sound using a scale from 1 (*pleasant*) to 5 (*unpleasant*) with midpoint marker (i.e., a rating of 3) represented as *neutral*. The white noise blast used in the aversive avoidance condition was rated as moderately to severely aversive (M = 4.41, SD = 0.95, range = 1–5), whereas the monetary reward sound was rated as pleasant‐to‐neutral (M = 2.39, SD = 1.07, range = 1–5). A paired samples *t‐*test indicated that these ratings were significantly different, *t*(99) = 14.40, *p* < 0.001. Sound ratings were not significantly related to the RewP in either the aversive avoidance (*r* = 0.08, *p* = 0.417) or monetary reward (*r* = 0.05, *p* = 0.607) conditions, respectively.

### 2.3 Reward Positivity

#### 2.3.1 RewP Condition by Trial Type Differences

A 2 (condition: safety, monetary) by 2 (trial type: win, loss) RM‐ANOVA was conducted with the signal averaged RewP components entered as dependent variables to evaluate the condition × trial type interaction across the full sample. As expected, results indicated a significant effect of trial type [*F*(1, 101) = 67.62, *p* < 0.001], indicating greater activity on winning trials (M = 12.26, SD = 6.70) relative to the losing trials (M = 9.90, SD = 6.73) when averaged across conditions. A significant condition [*F*(1, 101) = 12.37, *p* < 0.001] effect showed that greater overall neural activity in the monetary condition (M = 11.89, SD = 7.02) compared to the aversive avoidance condition (M = 10.28, SD = 6.88) regardless of trial type. A significant condition × trial type interaction [*F*(1, 101) = 4.29, *p* = 0.041] indicated that there was a larger difference between avoidance win (M = 11.71, SD = 7.18) relative to avoidance loss (i.e., pending noise blast; M = 8.86, SD = 7.10) than the difference between monetary reward win (M = 12.82, SD = 7.19) and monetary reward loss (M = 10.95, SD = 7.33).

#### 2.3.2 RM‐ANOVA Testing Differential RewP Responding by DI

A 2 (condition: safety, monetary) by 2 (trial type: win, loss) RM‐ANCOVA was conducted with the signal averaged components entered as dependent variables and DII total, DASS depression, DASS anxiety, and trauma history entered as independent variables to evaluate the hypothesized DII × condition × trial type interaction. Results indicated non‐significant DI × trial type [*F*(1, 97) = 0.32, *p* = 0.574] and DI × condition [*F*(1, 97) = 2.81, *p* = 0.097] interactions but a significant DI × trial type × condition interaction [*F*(1, 97) = 5.13, *p* = 0.026] did emerge indicating that DI significantly modulated differential responding across trial types and conditions (see Table 2).

**TABLE 2:** Repeated Measures Analysis of Variance Testing Differential RewP and Feedback P300 Responses Across Conditions.

DV: RewP	SS	df	MS	*F*	*p*	*ηp* ^2^
Condition	72.09	1	72.09	3.44	0.067	0.034
Condition × DII	58.75	1	58.75	2.81	0.097	0.028
Condition × DASS Anxiety	65.41	1	65.41	3.13	0.080	0.031
Condition × DASS Depression	0.68	1	0.68	0.03	0.857	0.000
Condition × LEC‐5 Trauma Load	19.36	1	19.36	0.93	0.339	0.009
Error (Condition)	2030.57	97	20.93			
Trial Type	93.83	1	93.83	11.13	0.001	0.103
Trial Type × DII	2.68	1	2.68	0.32	0.574	0.003
Trial Type × DASS Anxiety	1.27	1	1.27	0.15	0.698	0.002
Trial Type × DASS Depression	3.34	1	3.34	0.40	0.530	0.004
Trial Type × LEC‐5 Trauma Load	2.55	1	2.55	0.30	0.584	0.003
Error (Trial Type)	817.47	97	8.43			
Condition × Trial Type	4.30	1	4.30	0.78	0.380	0.008
Condition × Trial Type × DII	28.42	1	28.42	5.13	0.026	0.050
Condition × Trial Type × DASS Anxiety	16.83	1	16.83	3.04	0.084	0.030
Condition × Trial Type × DASS Depression	7.98	1	7.98	1.44	0.233	0.150
Condition × Trial Type × LEC‐5 Trauma Load	0.20	1	0.20	0.04	0.851	0.000
Error (Condition × Trial Type)	536.91	97	5.54			

DV: Feedback P300	SS	df	MS	*F*	*p*	*ηp* ^2^
Condition	67.14	1	67.14	3.55	0.063	0.035
Condition × DII	152.08	1	152.08	8.04	0.006	0.077
Condition × DASS Anxiety	143.73	1	143.73	7.60	0.007	0.073
Condition × DASS Depression	0.34	1	0.34	0.02	0.893	0.000
Condition × LEC‐5 Trauma Load	16.38	1	16.38	0.87	0.354	0.009
Error (Condition)	1834.51	97	18.91			
Trial Type	13.08	1	13.08	2.63	0.108	0.026
Trial Type ×DII	13.16	1	13.16	2.64	0.107	0.027
Trial Type × DASS Anxiety	17.23	1	17.23	3.46	0.066	0.034
Trial Type × DASS Depression	0.14	1	0.14	0.03	0.867	0.000
Trial Type × LEC‐5 Trauma Load	8.69	1	8.69	1.75	0.190	0.018
Error (Trial Type)	482.99	97	4.98			
Condition × Trial Type	2.58	1	2.58	0.57	0.450	0.006
Condition × Trial Type × DII	9.87	1	9.87	2.20	0.141	0.022
Condition × Trial Type × DASS Anxiety	5.67	1	5.67	1.26	0.264	0.013
Condition × Trial Type × DASS Depression	0.74	1	0.74	0.17	0.686	0.002
Condition × Trial Type × LEC‐5 Trauma Load	20.95	1	20.95	4.67	0.033	0.046
Error (Condition × Trial Type)	434.81	97	4.48			

Abbreviations: DASS, Depression, Anxiety and Stress Scale; DII, Distress Intolerance Index; LEC‐5, Life Events Checklist for DSM‐5.

#### 2.3.3 Association of DI With ΔAvoidance RewP

**FIGURE 1:**
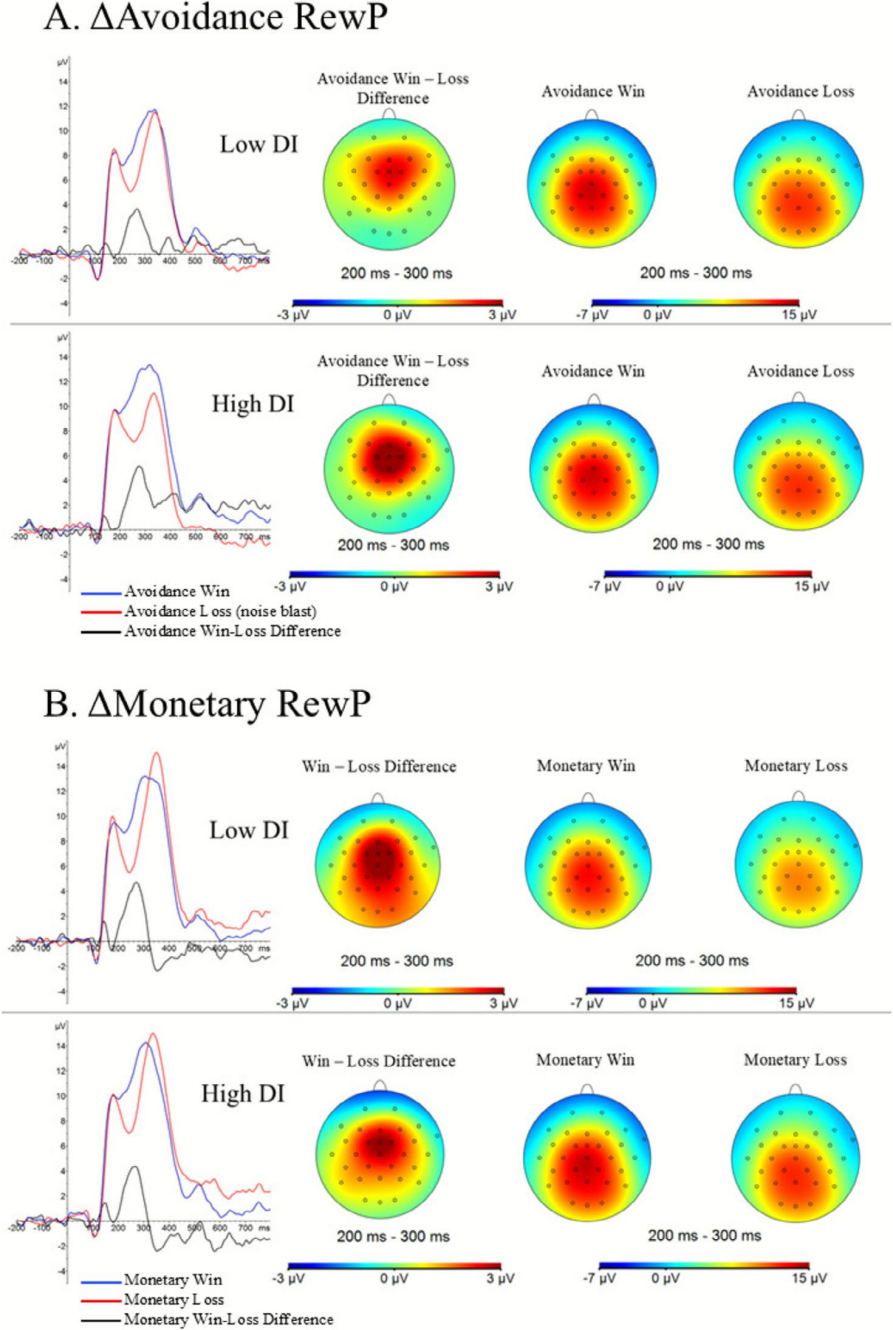
Waveforms and topographical maps depicting the signal‐averaged ΔAvoidance RewP and ΔMonetary RewP among those with high and low distress intolerance (DI). Panel A: Individuals with high DI, relative to low DI, demonstrate a larger ΔAvoidance RewP reflecting greater reward‐related brain activity to feedback indicating the avoidance of an aversive stimulus. Panel B: DI was not associated with the ΔMonetary RewP, indicating specificity of the DI construct to avoidance‐related neural activation. Signal averaged waveforms were indexed at FCz. DI, distress Intolerance. RewP, reward positivity. Avoidance win = feedback indicating successful aversive stimulus avoidance. Avoidance loss = feedback indicating the pending administration of an aversive stimulus. Monetary win = feedback indicating monetary gain. Monetary loss = feedback indicating monetary loss. Median split figures are for presentation only; all data were analyzed using continuous data.

The significant DI × condition × trial type interaction was further probed using hierarchical multiple linear regressions to test the directionality of the observed effects. The unstandardized residualized difference score (safety win relative to safety loss) was entered as the dependent variable and DI and covariates were entered as independent variables. State anxiety during the aversive avoidance blocks was entered as a covariate in the second step to determine if the effects were accounted for by state anxiety. Results indicated that DI was significantly associated with a greater ΔAvoidance RewP (*β* = 0.32, *t* = 2.26, *p* = 0.026) even when stringently accounting for related psychopathology (see Table 3 and Figure 1). Moreover, DI remained significantly associated with the ΔAvoidance RewP (*β* = 0.29, *t* = 2.08, *p* = 0.040) even when self‐reported state anxiety during the aversive avoidance blocks was included in the model.

**TABLE 3:** Hierarchical Multiple Linear Regressions Testing Differential RewP Across Conditions.

	IV	*β*	*t*	*p*	*r* ^2^	VIF
**DV: ΔAvoidance RewP**						
Step 1	DII Total	0.32	2.26	0.026	0.048	2.09
	DASS Anxiety	−0.14	−0.93	0.354	0.008	2.42
	DASS Depression	0.11	0.84	0.402	0.006	1.79
	LEC‐5 Trauma Load	−0.07	−0.63	0.529	0.004	1.10
Step 2	DII Total	0.29	2.08	0.040	0.040	2.12
	DASS Anxiety	−0.19	−1.22	0.228	0.014	2.52
	DASS Depression	0.08	0.64	0.526	0.004	1.82
	LEC‐5 Trauma Load	−0.07	−0.72	0.471	0.005	1.11
	Avoidance Condition State Anxiety	0.17	1.44	0.152	0.020	1.40
**DV: Avoidance win RewP**						
Step 1	DII Total	0.32	2.24	0.027	0.049	2.09
	DASS Anxiety	−0.16	−1.02	0.310	0.010	2.42
	DASS Depression	−0.16	−1.22	0.226	0.014	1.79
	LEC‐5 Trauma Load	−0.08	−0.72	0.471	0.005	1.10
Step 2	DII Total	0.32	2.20	0.031	0.048	2.12
	DASS Anxiety	−0.16	−1.03	0.307	0.010	2.52
	DASS Depression	−0.16	−1.22	0.225	0.015	1.82
	LEC‐5 Trauma Load	−0.08	−0.73	0.468	0.005	1.11
	Avoidance Condition State Anxiety	0.02	0.16	0.873	< 0.001	1.40
**DV: Avoidance loss RewP**						
Step 1	DII Total	0.18	1.29	0.202	0.016	2.09
	DASS Anxiety	−0.10	−0.65	0.520	0.004	2.42
	DASS Depression	−0.26	−1.98	0.050	0.038	1.79
	LEC‐5 Trauma Load	−0.05	−0.48	0.636	0.002	1.10
Step 2	DII Total	0.20	1.36	0.178	0.018	2.12
	DASS Anxiety	−0.08	−0.49	0.628	0.002	2.52
	DASS Depression	−0.25	−1.86	0.066	0.034	1.82
	LEC‐5 Trauma Load	−0.05	−0.43	0.669	0.002	1.11
	Avoidance Condition State Anxiety	−0.08	−0.70	0.489	0.005	1.40
**DV: ΔMonetary RewP**						
Step 1	DII Total	−0.11	−0.73	0.467	0.005	2.09
	DASS Anxiety	0.24	1.52	0.131	0.023	2.42
	DASS Depression	−0.10	−0.75	0.457	0.006	1.79
	LEC‐5 Trauma Load	−0.08	−0.76	0.447	0.006	1.10
Step 2	DII Total	−0.12	−0.81	0.420	0.007	2.10
	DASS Anxiety	0.22	1.38	0.171	0.019	2.46
	DASS Depression	−0.12	−0.90	0.373	0.008	1.84
	LEC‐5 Trauma Load	−0.06	−0.57	0.567	0.003	1.14
	Monetary Condition State Anxiety	0.11	0.96	0.340	0.009	1.23
**DV: Monetary gain RewP**						
Step 1	DII Total	0.05	0.36	0.723	0.001	2.09
	DASS Anxiety	0.12	0.74	0.461	0.005	2.42
	DASS Depression	−0.22	−1.66	0.101	0.028	1.79
	LEC‐5 Trauma Load	−0.03	−0.25	0.800	0.001	1.10
Step 2	DII Total	0.05	0.33	0.744	0.001	2.10
	DASS Anxiety	0.11	0.69	0.491	0.005	2.46
	DASS Depression	−0.23	−1.67	0.098	0.029	1.84
	LEC‐5 Trauma Load	−0.02	−0.20	0.845	< 0.001	1.14
	Monetary Condition State Anxiety	0.03	0.28	0.779	0.001	1.23
**DV: Monetary loss RewP**						
Step 1	DII Total	0.13	0.87	0.387	0.008	2.09
	DASS Anxiety	0.00	−0.03	0.980	0.000	2.42
	DASS Depression	−0.21	−1.55	0.125	0.024	1.79
	LEC‐5 Trauma Load	0.02	0.15	0.878	0.000	1.10
Step 2	DII Total	0.13	0.88	0.380	0.008	2.10
	DASS Anxiety	0.00	0.01	0.994	< 0.001	2.46
	DASS Depression	−0.20	−1.48	0.143	0.022	1.84
	LEC‐5 Trauma Load	0.01	0.11	0.915	< 0.001	1.14
	Monetary Condition State Anxiety	−0.03	−0.24	0.814	0.001	1.23
**DV: DII total**						
Step 1	ΔAvoidance RewP	0.29	2.87	0.005	0.080	1.06
	ΔMonetary RewP	−0.08	−0.83	0.410	0.010	1.06
Step 2	ΔAvoidance RewP	0.17	2.35	0.021	0.026	1.12
	ΔMonetary RewP	−0.11	−1.48	0.143	0.010	1.11
	DASS Anxiety	0.53	5.49	< 0.001	0.143	1.98
	DASS Depression	0.15	1.63	0.107	0.013	1.83
	LEC‐5 Trauma Load	0.04	0.48	0.633	0.001	1.18
	Avoidance Condition State Anxiety	0.08	0.80	0.423	0.003	1.83
	Monetary Condition State Anxiety	0.03	0.36	0.722	0.001	1.54

Abbreviations: ΔAvoidance RewP, unstandardized residualized difference score of Avoidance Win relative to Avoidance Loss; DASS, Depression, Anxiety and Stress Scale; DII, Distress Intolerance Index; LEC‐5, Life Events Checklist for DSM‐5; ΔMonetary RewP, unstandardized residualized difference score of Monetary Win relative to Monetary Loss; State anxiety, Self‐reported state anxiety during each respective condition.

Additional regressions showed that DI significantly predicted the reward‐related brain activity on the avoidance win trials (*β* = 0.32, *t* = 2.24, *p* = 0.027) but not on the avoidance loss trials indicating a pending aversive stimulus (*β* = 0.18, *t* = 1.29, *p* = 0.202), indicating that the observed association between DI and the ΔAvoidance RewP appears to be driven by greater brain activity to feedback indicating safety from an aversive stimulus (see Table 3 and Figure 1). No evidence of multicollinearity was noted (VIF's < 2.52; see Table 3).

#### 2.3.4 Association of DI With ΔMonetary RewP

The significant DI × condition × trial type interaction was further probed using hierarchical multiple linear regressions to test the directionality of the observed effects. The unstandardized residualized difference score (monetary win relative to monetary loss) was entered as the dependent variable, and DI and covariates were entered as independent variables. State anxiety during the monetary reward blocks was entered as a covariate in the second step to determine if the effects were accounted for by state anxiety. Results indicated that DI was not significantly associated with a greater ΔMonetary RewP (*β* = −0.11, *t* = −0.73, *p* = 0.467; see Table 3 and Figure 1). No evidence of multicollinearity was noted (VIF's < 2.52; see Table 3).

#### 2.3.5 Unique Associations of ΔAvoidance RewP on DI When Accounting for ΔMonetary RewP

Additional hierarchical linear regressions were conducted to test the relative associations of DI with the ΔAvoidance RewP and ΔMonetary RewP. In the first step, DI was significantly related to a greater ΔAvoidance RewP (*β* = 0.29, *t* = 2.87, *p* = 0.005) but not the ΔMonetary RewP (*β* = −0.08, *t* = −0.83, *p* = 0.410). DI remained significantly associated with the ΔAvoidance RewP (*β* = 0.17, *t* = 2.35, *p* = 0.021) even when psychopathology covariates (DASS anxiety, DASS depression, and trauma load) and state anxiety during each condition were added to the model (VIFs < 1.97). DI was not associated with ΔMonetary RewP (*p*'s > 0.143; see Table 3). No evidence of multicollinearity was noted (VIF's < 1.98; see Table 3).

### 2.4 Feedback‐Locked P300

#### 2.4.1 Feedback P300 Condition by Trial Type Differences

A 2 (condition: safety, monetary) by 2 (trial type: win, loss) RM‐ANOVA was conducted with the signal averaged Feedback P300 components entered as dependent variables to evaluate the condition × trial type interaction across the full sample. Results indicated a significant effect of condition [*F*(1, 101) = 9.28, *p* = 0.003], indicating greater neural activity in the monetary condition (M = 14.38, SD = 6.35) compared to the aversive avoidance condition (M = 13.02, SD = 6.20) across trial types. Non‐significant effects of trial type [*F*(1, 101) = 0.84, *p* = 0.361] and condition × trial type [*F*(1, 101) = 2.43, *p* = 0.122] indicated that the feedback‐locked P300 was not modulated by trial type in either condition when examined across the full sample.

#### 2.4.2 RM‐ANOVA Testing Differential Feedback P300 Responding by DI

**FIGURE 2:**
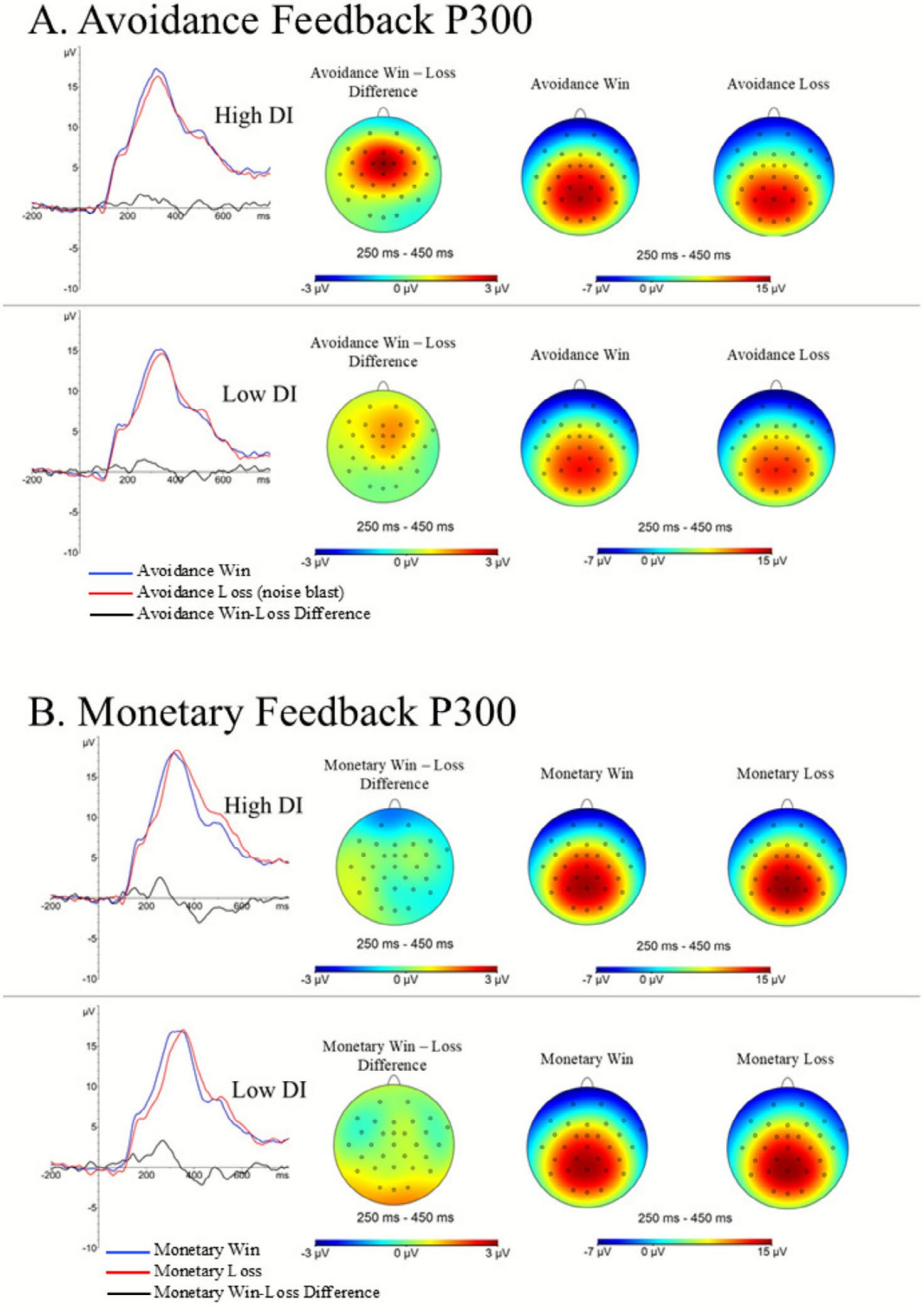
Waveforms and topographical maps depicting the signal‐averaged Avoidance and Monetary Feedback‐locked P300 components among those with high and low distress intolerance (DI). Panel A: Individuals with high DI, relative to low DI, demonstrate a larger Avoidance Feedback P300 across trial types. Panel B: DI was not associated with the Monetary Feedback P300. DI , distress Intolerance. Feedback P300 = feedback‐locked P300 waveform signal averaged from 250 to 450 ms at Pz. Avoidance win = feedback indicating successful aversive stimulus avoidance. Avoidance loss = feedback indicating the pending administration of an aversive stimulus. Monetary win = feedback indicating monetary gain. Monetary loss = feedback indicating monetary loss. Median split figures are for presentation only; all data were analyzed using continuous data.

A 2 (condition: safety, monetary) by 2 (trial type: win, loss) RM‐ANCOVA was conducted with the signal averaged feedback‐locked P300 components entered as dependent variables and DII total, DASS depression, DASS anxiety, and trauma history entered as independent variables to evaluate the hypothesized DII × condition × trial type interaction. Results indicated a significant DI × condition [*F*(1, 97) = 8.04, *p* = 0.006] interaction did emerge indicating that DI differentially modulated the feedback‐locked P300 across monetary reward and aversive avoidance conditions (see Table 2 and Figure 2). The DI × trial type [*F*(1, 97) = 2.64, *p* = 0.107] and DI × trial type × condition [*F*(1, 97) = 2.20, *p* = 0.141] interactions were non‐significant, indicating that DI did not modulate differential trial type responding either across or within conditions.

#### 2.4.3 Probing Association of DI With Feedback‐Locked P300 Across Conditions

The significant DI × Condition interaction was further probed using linear regressions to test the directionality of the observed effects. Given that the RM‐ANOVA showed that DI did not differentially modulate trial type (win/loss) responding, win/loss trials were collapsed across each condition and used as dependent variables. Results indicated that DI was significantly associated with the avoidance condition feedback‐locked P300 (β = 0.36, *t* = 2.57, *p* = 0.012) even when accounting for DASS anxiety (β = −0.06, *t* = −0.39, *p* = 0.697), DASS depression (β = −0.33, *t* = −2.49, *p* = 0.014), trauma load (β = −0.09, *t* = −0.86, *p* = 0.390), and avoidance task state anxiety (β = 0.03, *t* = 0.25, *p* = 0.805). Results indicated that DI was not associated with the monetary reward feedback‐locked P300 (β = 0.08, *t* = 0.57, *p* = 0.573) when accounting for DASS anxiety (β = 0.26, *t* = 1.64, *p* = 0.103), DASS depression (β = −0.29, *t* = −2.12, *p* = 0.036), trauma load (β = −0.04, *t* = −0.34, *p* = 0.734), and monetary task state anxiety (β = −0.06, *t* = −0.50, *p* = 0.616). No evidence of multicollinearity was noted (VIF's < 2.52; see Table 4).

**TABLE 4:** Hierarchical Multiple Linear Regressions Testing Differential Feedback P300 Across Conditions.

	IV	*β*	*t*	*p*	*r2*	*VIF*
**DV: Avoidance feedback P300**						
Step 1	DII Total	0.37	2.63	0.010	0.065	2.09
	DASS Anxiety	−0.05	−0.35	0.728	0.001	2.42
	DASS Depression	−0.32	−2.50	0.014	0.058	1.79
	LEC‐5 Trauma Load	−0.09	−0.85	0.396	0.007	1.10
Step 2	DII Total	0.36	2.57	0.012	0.062	2.12
	DASS Anxiety	−0.06	−0.39	0.697	0.001	2.52
	DASS Depression	−0.33	−2.49	0.014	0.059	1.82
	LEC‐5 Trauma Load	−0.09	−0.86	0.390	0.007	1.11
	Avoidance Condition State Anxiety	0.03	0.25	0.805	0.001	1.40

	IV	*β*	*t*	*p*	*r* ^2^	VIF
**DV: Monetary feedback P300**						
Step 1	DII Total	0.08	0.53	0.600	0.003	2.09
	DASS Anxiety	0.25	1.60	0.114	0.025	2.42
	DASS Depression	−0.30	−2.25	0.027	0.049	1.79
	LEC‐5 Trauma Load	−0.03	−0.25	0.800	0.001	1.10
Step 2	DII Total	0.08	0.57	0.573	0.003	2.10
	DASS Anxiety	0.26	1.64	0.103	0.027	2.46
	DASS Depression	−0.29	−2.12	0.036	0.045	1.84
	LEC‐5 Trauma Load	−0.04	−0.34	0.734	0.001	1.14
	Monetary Condition State Anxiety	−0.06	−0.50	0.616	0.003	1.23

	IV	*β*	*t*	*p*	*r* ^2^	VIF
**DV: DII total**						
Step 1	Avoidance Feedback P300	0.16	1.06	0.291	0.011	2.21
	Monetary Feedback P300	−0.05	−0.32	0.752	0.001	2.21
Step 2	Avoidance Feedback P300	0.33	3.22	0.002	0.047	2.31
	Monetary Feedback P300	−0.21	−1.99	0.050	0.018	2.35
	DASS Anxiety	0.53	5.49	< 0.001	0.137	2.08
	DASS Depression	0.22	2.42	0.017	0.027	1.85
	LEC‐5 Trauma Load	0.05	0.73	0.468	0.002	1.17
	Avoidance Condition State Anxiety	0.07	0.75	0.456	0.003	1.75
	Monetary Condition State Anxiety	0.02	0.20	0.840	0.000	1.55

Abbreviations: DII, Distress Intolerance Index; DASS, Depression, Anxiety and Stress Scale; LEC‐5, Life Events Checklist for DSM‐5; State anxiety, self‐reported state anxiety during each respective condition.

#### 2.4.4 Unique Associations of Avoidance Feedback‐Locked P300 on DI When Accounting for Monetary Feedback‐Locked P300

Additional hierarchical linear regressions were conducted to test the relative associations of DI with the avoidance feedback‐locked P300 and monetary feedback‐locked P300. Given that DI was not linked with differential trial type responding on the feedback‐locked P300, the win and loss trials were collapsed for each condition.^3^ In the first step, DI was not significantly related to a greater avoidance feedback‐locked P300 (*β* = 0.16, *t* = 1.06, *p* = 0.291) when accounting for only the monetary feedback‐locked P300 (*β* = −0.05, *t* = −0.32, *p* = 0.752). However, DI was significantly associated with a greater avoidance feedback‐locked P300 (*β* = 0.33, *t* = 3.22, *p* = 0.002) and reduced monetary feedback‐locked P300 (*β* = −0.21, *t* = −1.99, *p* = 0.050) when psychopathology (DASS anxiety, DASS depression, and trauma load) and state anxiety during each task condition were included in the model (VIF's < 2.35; see Table 4).

## 3 Discussion

Avoidance of aversive stimuli, and their repetition via negative reinforcement, is a basic adaptive mechanism that drives a host of behaviors across species (LeDoux et al. 2017). While negatively reinforced behaviors can be adaptive (e.g., putting on a seatbelt to turn off a reminder noise), such behaviors deployed in maladaptive situations can contribute to the development and maintenance of various forms of psychopathology. To the best of our knowledge, our findings are the first to demonstrate unique associations of an avoidance‐related construct with greater initial responses to avoidance feedback. Specifically, DI, a construct marked by the escape and avoidance of distressing emotions, is associated with an elevated RewP specifically when the “reward” is avoidance of an aversive stimulus (i.e., ΔAvoidance RewP) but not when receiving a monetary reward (ΔMonetary RewP). DI was also associated with a greater feedback‐locked P300 during the avoidance condition, regardless of trial type. These findings build on theoretical and empirical work linking DI with avoidance‐related mechanisms (e.g., Leyro et al. 2010; Vujanovic and Zegel 2020) by suggesting that those with DI experience avoidance as more rewarding. Moreover, this study has broad implications for understanding avoidance‐related disruptions across psychopathology through the lens of reward responsivity.

Demonstrating that DI is marked by greater reward responses to aversive stimulus avoidance sheds new light on the neurobehavioral processes associated with high DI and suggests that those with high DI may experience avoidance as more rewarding. Existing studies seeking to understand the negative reinforcement aberrations apparent in elevated DI have primarily relied on experimental paradigms in which persistence during a frustrating task is used as a behavioral indicator of individual differences to the tolerance of negative emotions (McHugh and Otto 2012). While these paradigms largely show the expected relations with relevant psychopathology, associations with self‐reported DI have been inconsistent (McHugh and Otto 2012). These discrepancies raise questions regarding contributing factors to self‐reported perceived DI and whether relief from distressing emotions sufficiently captures the construct. By leveraging the RewP (Proudfit 2015) to reflect reward responses to negatively reinforcing feedback (Albanese and Hajcak 2021; Mulligan and Hajcak 2018), the present study demonstrated that individuals with elevated DI may experience negative reinforcement as more rewarding than those with lower DI. Further, these differences were not better explained by aversiveness ratings of the stimuli, valence‐agnostic general reward responsivity, relevant psychopathology (i.e., anxiety, depression), or trauma history. This suggests that high DI may be marked by both “push” and “pull” factors in that individuals with elevated DI experience distressing emotions as less tolerable and are pulled towards avoidance behaviors that are subsequently experienced as more rewarding. Taken together, this reconceptualization may hold significant promise for not only understanding the DI construct but also related psychopathology that is linked with DI and negative reinforcement aberrations.

These findings also have broader implications for reward‐related research. The present study showed a modest correlation (*r* = 0.23) between the ΔAvoidance RewP and ΔMonetary RewP, as well as unique effects of the ΔAvoidance RewP with an important, avoidance‐related risk factor. This pattern implies that varying the type of reward elicits RewP components that have both shared and unique variance and dovetails with other work leveraging the RewP to understand the relative influences of cigarette and non‐cigarette rewards among smokers (Baker et al. 2016). Moreover, the correlations between the feedback‐locked P300 conditions (*r*'s > 0.68) and between win/loss trials within conditions (*r*'s > 0.88) suggest that the P300 may track unique reward‐related differences with less specificity than the RewP. This study adds to a growing body of work showing that task manipulations of the reward types can be leveraged to study both general (e.g., broad anhedonia in depression; Proudfit 2015) and specific (e.g., cigarettes, avoidance; Albanese and Hajcak 2021; Baker et al. 2016) reward disruptions and suggests that the RewP may provide greater specificity of reward valuation, though more research is needed to confirm this. Taken together, there is mounting evidence that the reward valence and type is critical to understanding reward‐related dysfunction across psychopathology, and that the ΔAvoidance RewP represents a promising avenue for understanding aberrations of negative reinforcement that underlie many forms of psychopathology.

Illuminating a novel neural mechanism that may contribute to DI also has important clinical implications. DI has been shown to be malleable (Heiland and Veilleux 2024; Macatee and Cougle 2015; Reese et al. 2019), and reductions in DI yield improvements in related psychopathology (Bornovalova et al. 2012; Macatee et al. 2021, 2022). The identification of an additional neural mechanism that may contribute to DI may provide another target by which to reduce DI, such as through transcranial magnetic stimulation (TMS), which has been shown to be able to modulate reward‐related brain activity by increasing appetitive rewards (Baker et al. 2017). More work is needed to evaluate whether aversive avoidance reward responding could be reduced. In addition, the knowledge that aversive avoidance may be experienced as more rewarding by those with elevated DI may also inform the refinement of existing DI interventions that focus largely on increasing the perceived or actual tolerance of negative emotionality.

There are several important limitations to consider as well. First, the reliabilities of the ΔAvoidance RewP and ΔMonetary RewP were both relatively low, especially the ΔMonetary RewP, which could have impacted the observed effect sizes (Clayson et al. 2021; Heindorf et al. 2025) thereby potentially suppressing a relation between DI and the ΔMonetary RewP. Future research should potentially use greater trial numbers to enhance reliability, which may be particularly important for the monetary doors condition. Second, while we observed larger activity overall in the monetary reward condition across both win and loss trials, the aversive avoidance reward condition showed a larger trial type differentiation between winning (i.e., safety from the noise blast) and losing (i.e., pending administration of the noise blast). This differs from other studies (e.g., Mulligan and Hajcak 2018), which found no overall differences in the RewP. This could be related to the difference in the aversive stimuli; the present study used an aversive noise blast with a static intensity across all participants whereas Mulligan and Hajcak (2018) used a mild electrical shock, the intensity of which was set by each participant. Further, mild electric shock and loud sounds could represent conceptually distinct aversive stimuli that elicit differential responding across participants, with the threat‐of‐shock possibly tapping into variance also associated with pain tolerance. Additional research is needed to compare the RewP when using an array of aversive stimuli and aversive/appetitive unconditioned stimulus timing (e.g., immediate vs. delayed) to inform this growing area of work.

Further, this study used a cross‐sectional design, precluding causal inferences. Future work should evaluate the bi‐directional influences of DI and the Δ avoidance RewP to determine if perceived DI influences reward responses to safety signals or vice versa. Third, only self‐reported DI was assessed in the current study. Considering past research has shown inconsistent associations between self‐report and behavioral measures of the DI construct, future research should test whether a greater Δ avoidance RewP also predicts latency to quit distressing tasks (Lejuez et al. 2003). Fourth, the sample was predominantly comprised of young adults who mostly identified as female and Caucasian/White adults. While samples of emerging adults are well‐suited to research on risk factors such as DI, future efforts should recruit more diverse samples across racial, ethnic, age, and other demographic variables. Finally, the current study was based on a modest sample size and a nonclinical sample with relatively low scores on self‐report indices of anxiety, depression, and DI; further examination of these ERPs and their relative influences on DI and related psychopathology among clinical and/or treatment‐seeking samples is warranted.

Taken together, the present study is the first, to our knowledge, to demonstrate the association between self‐reported DI and neural reward responses to aversive stimulus avoidance. Results suggest that DI is linked with a larger ΔAvoidance RewP, potentially reflecting a stronger neural response to negatively reinforcing feedback. These findings not only expand our understanding of the DI construct but are also among the first to demonstrate the utility of the ΔAvoidance RewP for understanding individual differences in negative reinforcement aberrations across affective risk factors and disorders (Albanese and Hajcak 2021; Preston et al. 2022). Future work should incorporate other neurocognitive mechanisms to contribute to DI, including facets of cognitive control (Andrés et al. 2021; Macatee, Albanese, Clancy, et al. 2018) and prefrontal functional connectivity (Daughters et al. 2017) and further examine whether the ΔAvoidance RewP and its association with avoidance‐related constructs is modulated by the immediacy/delay of the threat, magnitude of the threat, and self‐reported affective reactions to the avoidance feedback (c.f., *relief*).

## Author Contributions

**Brian J. Albanese:** conceptualization, investigation, writing – original draft, funding acquisition, methodology, validation, visualization, writing – review and editing, software, formal analysis, project administration, data curation, supervision, resources. **Daniela Porro:** writing – review and editing, writing – original draft, project administration. **David H. Johnson:** writing – review and editing, writing – original draft, project administration. **Bryce K. Clausen:** writing – original draft, writing – review and editing, project administration. **Anka A. Vujanovic:** writing – review and editing. **Richard J. Macatee:** writing – review and editing.

## Funding

This work was supported by U.S. Department of Health and Human Services, National Institutes of Health, and National Institute of Mental Health, L30MH127669.

## Conflicts of Interest

The authors declare no conflicts of interest.

## Supporting information


**Table S1:** Descriptive Statistics and Correlations for all Study Variables.

**Figure S1:** Violin plots showing the distributions for the and ΔMonetary RewP (left) and ΔAvoidance RewP (right).
**Figure S2:** Violin plot showing the distribution for the Distress Intolerance Index (DII).
**Figure S3:** Scatterplots and trend lines for the associations between Distress Intolerance Index (DII) and the ΔMonetary RewP (left) and ΔAvoidance RewP (right).

## Data Availability

The data that support the findings of this study are available on request from the corresponding author. The data are not publicly available due to privacy or ethical restrictions.

## Endnotes

^1^
Analyses were also completed using subtraction‐based scoring methods (e.g., Clayson et al. 2021). Subtraction‐based scores were highly correlated with the residualized difference scores for both the ΔAvoidance RewP (*r* = 0.97, *p* < 0.001) and the ΔMonetary RewP (*r* = 0.94, *p* < 0.001) and no notable differences in the patterns of effects with DI emerged.

^2^
One participant represented an outlier on the ∆Monetary RewP. Results were consistent when this subject was removed. Therefore, this participant was retained for all analyses.

^3^
Follow‐up analyses with the constituent feedback‐locked P300 for win and loss trials in each condition were also conducted; elevated variance inflation factor (VIF's > 5) suggested multicollinearity. Considering that the results indicated that DI did not modulate trial type differential responding, results were presented with the trial types collapsed across condition.
