# Supplementary material for: Distress Intolerance Is Associated With a Greater Reward Positivity to Aversive Avoidance Feedback
Source: Psychophysiology. 2026 Feb 4;63(2):e70236. doi: 10.1111/psyp.70236 (PMC12869353; doi:10.1111/psyp.70236)
Supplement: Supplementary file 2 — Figure S1: Violin plots showing the distributions for the and ΔMonetary RewP (left) and ΔAvoidance RewP (right). Figure S2: Violin plot showing the distribution for the Distress Intolerance Index (DII). Figure S3: Scatterplots and trend lines for the associations between Distress Intolerance Index (DII) and the ΔMonetary RewP (left) and ΔAvoidance RewP (right). [file PSYP-63-e70236-s001.docx]

**Supplemental Materials**


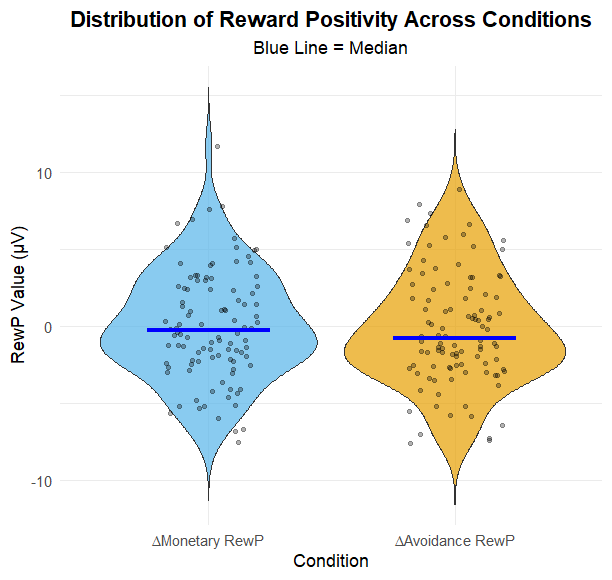


*Figure S1:* Violin plots showing the distributions for the and ΔMonetary RewP (left) and ΔAvoidance RewP (right). RewP = Reward Positivity. µV = amplitude in microvolts.


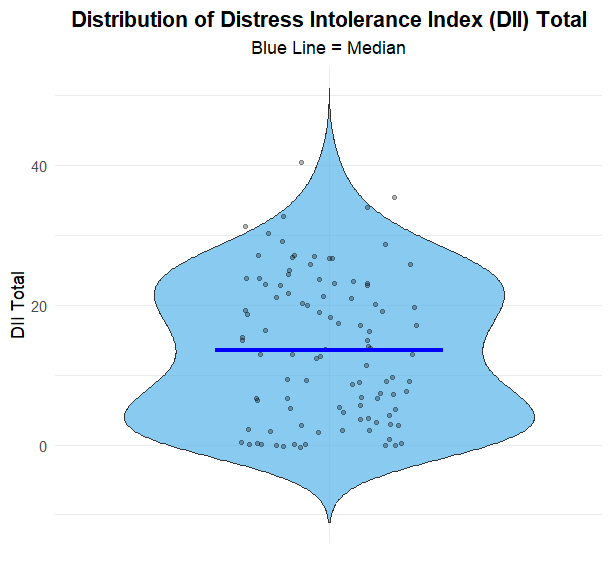


*Figure S2:* Violin plot showing the distribution for the Distress Intolerance Index (DII).


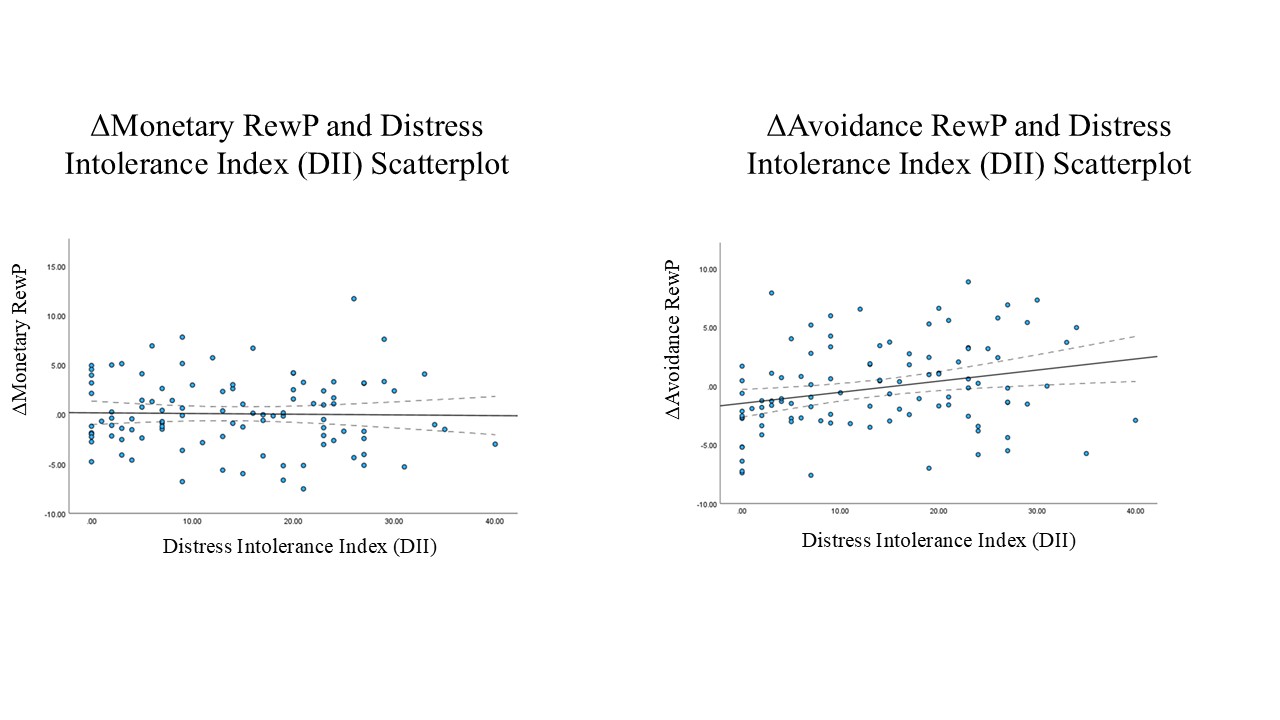


*Figure S3:* Scatterplots and trend lines for the associations between Distress Intolerance Index (DII) and the ΔMonetary RewP (left) and ΔAvoidance RewP (right).
